# Cryo-EM structure of human AQP11 reveals a trimeric architecture with a large pore

**DOI:** 10.1126/sciadv.aeb5769

**Published:** 2026-01-30

**Authors:** Shota Suzuki, Akiko Kamegawa, Daisuke Kozai, Kouki Nishikawa, Katsumasa Irie, Yoshinori Fujiyoshi

**Affiliations:** ^1^ Advanced Research Initiative, Institute of Integrated Research, Institute of Science Tokyo, 1-5-45 Yushima Bunkyo-ku, Tokyo 113-8501, Japan.; ^2^ Joint Research Course for Advanced Biomolecular Characterization, Faculty of Agriculture, Tokyo University of Agriculture and Technology, 3-5-8 Saiwai-cho, Fuchu, Tokyo 183-8509, Japan.; ^3^ Department of Biophysical chemistry School of Pharmaceutical Science, Wakayama Medical University, Wakayama 640-8156, Japan.

## Abstract

Aquaporin-11 (AQP-11) is an endoplasmic reticulum-localized water channel essential for renal development. Its structure and the molecular basis of its transport properties remained unknown. We analyzed the human AQP11 structure under cryo–electron microscopy at 2.3 Å resolution, revealing a trimeric architecture compared with other known tetrameric AQPs and a topology comprising seven transmembrane helices (Hs), including an additional N-terminal helix (H0). The channel pore is broader and more hydrophobic than that of canonical AQPs, and features a unique structure surrounding an Asn-Pro-Cys (NPC) sequence instead of the typical Asn-Pro-Ala (NPA) motif. These features provide a structural framework through which water and other small solutes can permeate AQP11. Our findings provide a blueprint for designing specific inhibitors to investigate the physiologic functions of AQP11.

## Teaser

The trimeric structure of AQP11 forms a wide pore for water and solutes and offers a previously unknown drug target.

## INTRODUCTION

**Fig. 1.**
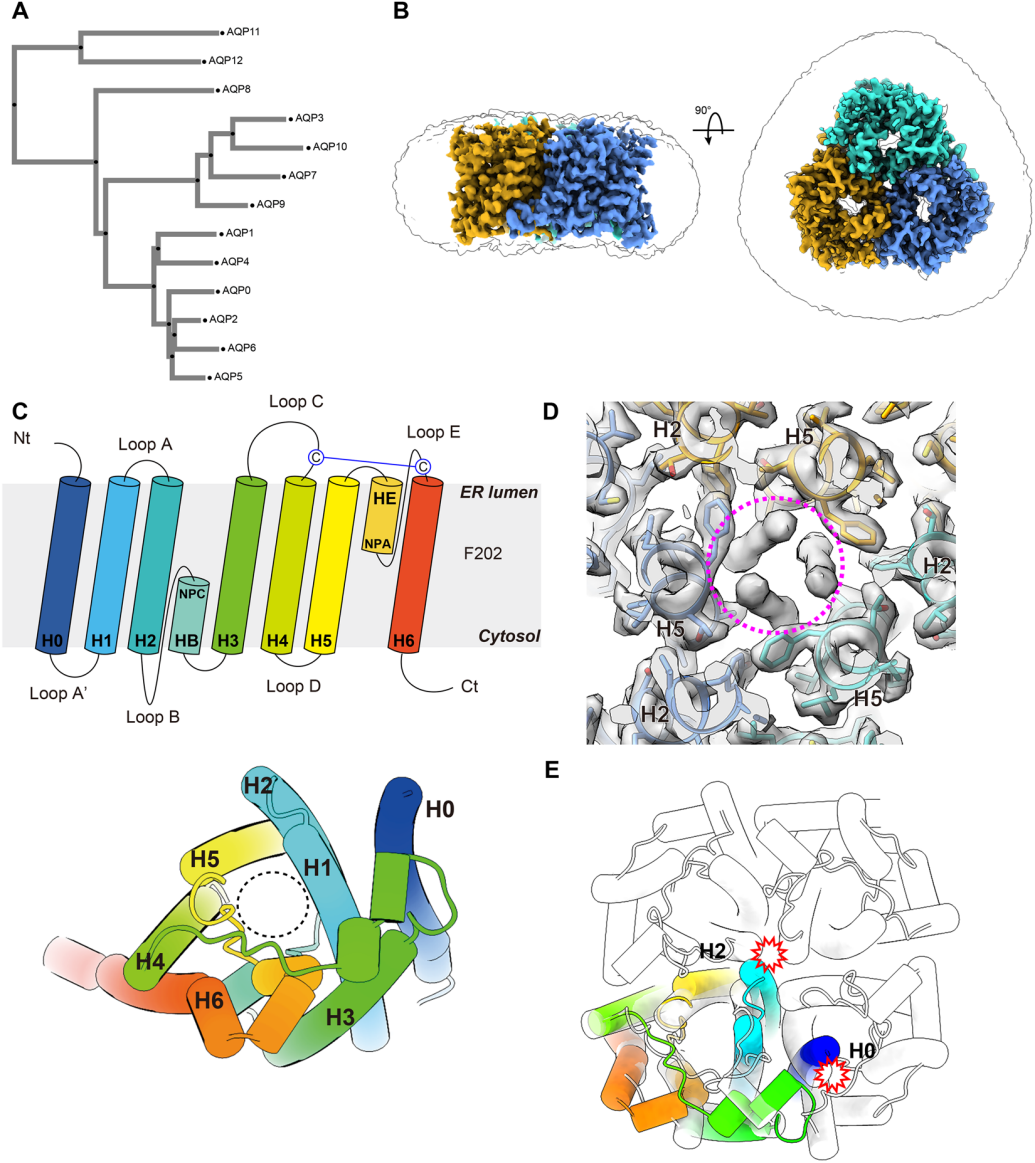
Overall structure of hAQP11. (**A**) A phylogenetic tree showing the relationship of the 13 water channel proteins in mammals (AQP0-AQP12). The phylogenetic tree was generated using MAFFT version 7. (**B**) Cryo-EM density map of hAQP11, showing side and top views. Each monomer is colored differently: green, blue, and yellow, arranged in a clockwise direction. (**C**) Schematic topology of a hAQP11 monomer colored in a rainbow. The NPA motifs at the HB and HE short helices are indicated as NPC/NPA (upper). A disulfide forming Cys residues are indicated. Top-view cartoon model of the AQP11 monomer is shown (lower). The helix numbers are indicated. A black dotted circle indicates the channel pore. (**D**) Central unidentified densities are shown and indicated as a purple dotted circle. (**E**) Overlay of the tetrameric structure of AQP4 and the AQP11 protomer. H0 and H2 clash at the tetrameric interface.

Water transport across biologic membranes is a fundamental process of life primarily governed by the aquaporin (AQP) family of channel proteins (*1*, *2*). AQPs facilitate rapid and selective permeation of water and, in some cases, small neutral solutes such as glycerol, and thereby play pivotal roles in a wide range of physiologic processes, from renal water reabsorption to neural signaling. The AQP family is broadly divided into three major subfamilies: strictly water-selective orthodox water channel AQPs (AQP0, 1, 2, 4, and 5); aquaglyceroporins (AQP3, 7, 9, and 10), which also transport glycerol and other small solutes (*3*, *4*); and a less-characterized subfamily consisting of AQP11 and AQP12 (Fig. 1A). As the third subfamily of AQPs exhibits less than 20% sequence homology with the other subfamilies, it is presumed that these AQPs have unique functional characteristics (*5*).

Unlike most AQPs, which locate in the plasma membrane, AQP11 primarily locates in the endoplasmic reticulum (ER) membrane (*6*). The physiologic importance of AQP11 is highlighted by the finding that knockout mice develop fatal polycystic kidneys shortly after birth, revealing its critical role in early renal development and function (*6*, *7*). Despite this crucial role, the precise transport function of AQP11 has remained unclear and controversial. Although initially proposed to be a water channel, studies have yielded conflicting results regarding its water permeability, and the potential transport of glycerol, hydrogen peroxide (H_2_O_2_), or other solutes has also been suggested (*8*–*12*). A distinguishing feature of AQP11 is its Asn-Pro-Cys (NPC) motif, which replaces the first highly conserved Asn-Pro-Ala (NPA) motif that is crucial for proton exclusion and water selectivity in traditional AQPs (*13*, *14*). Because of the absence of high-resolution structural information, the structural and functional implications of this substitution, as well as the molecular basis of the subcellular localization and substrate specificity of AQP11, have remained unclear. Previous efforts to determine its structure were hindered by the intrinsic instability of AQP11 and the challenges in producing and purifying sufficient quantities of the protein.

Understanding the molecular architecture of AQP11 is important for elucidating its unique transport mechanism and physiologic roles. Here, we report the cryo–electron microscopy (cryo-EM) structure of human AQP11 (hAQP11), embedded in lauryl maltose neopentyl glycol (LMNG) at 2.3 Å resolution. The structure exhibits a trimeric assembly distinct from any other AQPs and features an additional transmembrane helix at the N terminus. The pore architecture also shows notable features, including a specific conformation surrounding the NPC motif at the canonical NPA motif and a far wider, more hydrophobic pathway than water-selective AQPs. These structural insights provide a molecular framework for understanding the permeability characteristics of hAQP11, shedding light on its potential role in transporting water and possibly other small solutes, such as H_2_O_2_, across the ER membrane. Furthermore, the structure of hAQP11 presents a basis for designing selective inhibitors to facilitate future physiologic investigations.

## RESULTS

### Overall structure of trimeric hAQP11

hAQP11 was successfully produced using the Expi293 BacMam expression system. In brief, Expi293F cells were infected with a baculovirus carrying a pFastBac Mam-derived expression vector. This vector encodes hAQP11 tagged with a C-terminal superfolder green fluorescent protein and an 8× His tag. hAQP11 formed a stable oligomer during purification, as confirmed by size exclusion chromatography (SEC) profiles (fig. S1A). hAQP11 was first solubilized in *n*-dodecyl-β-d-maltopyranoside (DDM), which was then exchanged with LMNG. For structural analysis, we utilized our eighth-generation cryo-EM system (G8), which is based on a JEM-Z320FHC electron microscope originally developed in collaboration with JEOL (*15*). Unexpectedly, two-dimensional (2D) class averages revealed that hAQP11 exists in a unique homotrimeric state. The absence of other oligomeric states further confirmed the sample’s homogeneity (fig. S1, B and C). To confirm that this trimeric state was not a detergent-specific artifact, we performed detergent screening using fluorescence-detection SEC (FSEC). This analysis showed that AQP11 consistently eluted as a stable, monodisperse peak at a size smaller than the AQP4 tetramer control, even when solubilized in various detergents (DDM, LMNG, glyco-diosgenin (GDN), *n*-decyl-β-d-maltopyranoside (DM), and *n*-octyl-β-d-glucopyranoside (OG) (fig. S2, A and B). Furthermore, computational predictions using AlphaFold 3 (AF3) (*16*) strongly supported this finding, modeling AQP11 as a high-confidence trimer (fig. S2C) but as an artifactual tetramer (fig. S2D), indicating the trimeric assembly is an intrinsic property of AQP11. The final reconstruction of hAQP11 embedded in an LMNG micelle yielded a cryo-EM map with an overall resolution of 2.3 Å (Fig. 1B, fig. S1, D to G, and table S1). The high-resolution EM map enabled unambiguous modeling of residues Ser^9^ to His^262^. Although the structure unexpectedly revealed a trimeric assembly of hAQP11, each monomer adopted an AQP-like fold comprising six transmembrane helices (H1 to H6) and five connecting loops (A to E) (Fig. 1C and fig. S3). Moreover, an additional helix (H0) was observed at the N terminus, connected to H1 via loop A’ (Fig. 1C and figs. S3 and S4A). This seven-transmembrane helical topology orients the N terminus toward the ER lumen and the C terminus toward the cytosol, a finding that revises conclusions drawn from previous cell biologic experiments (*12*) (Fig. 1C). The first NPC motif and the second NPA motif of hAQP11 are located at two short helices, HB and HE, in re-entrant loops B and E, respectively (Fig. 1C).

An unidentified density was detected in the central axis of the AQP11 trimer (Fig. 1D). The ER and cytoplasmic sides of the central pore of AQP11 are blocked by the packing of hydrophobic residues, making it unlikely for unassigned density to enter from the external environment. AQP5 and EcAQPZ are also known to have a similar lipid-like density (*17*, *18*). The insertion of lipids into oligomeric interfaces is not unprecedented and has been observed in other membrane proteins, such as a channel rhodopsin (*19*). This suggests that these unassigned molecules contribute to the structural integrity of the oligomer by stabilizing weak interprotomer interactions, thereby facilitating trimer assembly.

Canonical AQPs typically form tetramers. Superimposing an AQP11 protomer onto one from a representative tetrameric AQP reveals that the N-terminal helix of AQP11, H0, would cause steric hindrance with an adjacent protomer in the tetrameric assembly (Fig. 1E). Sequence alignment of AQPs revealed that AQP4, 7, 8, and 11 have longer N-terminal domains compared to other AQPs (fig. S4B). While the N-terminal region of AQP11 exhibits high hydrophobicity and can form H0, the same region in AQP4, 7, and 8 is rich in polar amino acid residues, suggesting that it may not form a transmembrane structure (fig. S4B). The alignment shows that an extended N-terminal region is indeed a common feature of the AQP11/12 subfamily. The N terminus of AQP12 is also rich in hydrophobic residues, similar to helix H0 of AQP11 (fig. S4B). However, these hydrophobic regions in AQP12 are significantly shorter, approximately half the length of the H0 helix. Because of this shorter length, it is unlikely that this region in AQP12 is long enough to form a stable, membrane-spanning helix comparable to H0 in AQP11. Therefore, on the basis of the sequence alignment alone, we cannot conclude that AQP12 shares the same H0 transmembrane helix.

To investigate the importance of the N terminus of AQP11, we generated a mutant lacking H0 and analyzed its oligomeric state by FSEC. The results showed that the H0-deletion mutant still formed a trimer, although its expression level was markedly reduced (fig. S4C). This experimental finding is strongly supported by our AF3 predictions. When the H0-deletion mutant was modeled, it resulted in low-confidence scores for the interprotomer interfaces for both the trimer (fig. S2E) and tetramer (fig. S2F) models. Furthermore, a similar decrease in expression was observed upon deletion of the flexible N-terminal segment (residues 1 to 8). “On the basis of these combined experimental and computational findings, we concluded that the N-terminal region of AQP11 plays an indispensable role in the stable expression of the entire protein, and trimeric assembly of AQP11 is intrinsically determined by specific protein-protein interfaces inherent to the core structure of the protomer.

### AQP11 forms a unique trimer with distinct features from canonical AQPs

**Fig. 2.**
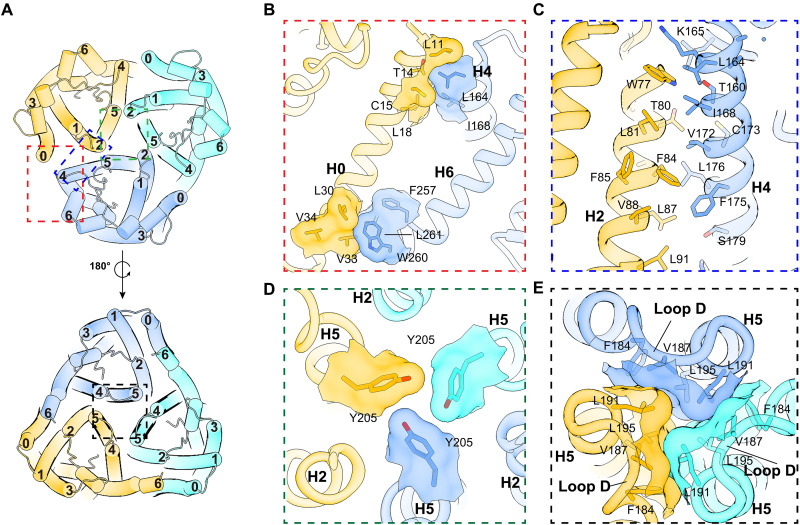
Oligomer interface. (**A**) The ER lumen view of hAQP11 (upper) and the cytosolic view (lower). Helical arrangements are shown as a cartoon model and colored as in Fig. 1B. (**B** to **E**) Enlarged illustration showing detailed interactions. Residues involved between different protomers are shown as sticks, overlapped with a transparent surface display of the model. (B) View of the red frame in (A) parallel to the membrane surface. H0 and H6 form hydrophobic interactions on the cytoplasmic side, and H0 and H4 form hydrophobic interactions on the ER side. (C) View of the blue-framed area in (A) parallel to the membrane surface. H2 and H4 between adjacent protomers form extensive hydrophobic interactions. (D) Enlarged view of the green frame in (A). The positioning of Tyr^205^ occludes the trimer’s central axis on the cytoplasmic side. (E) Enlarged view of the black frame in (A). Interactions with hydrophobic residues in loop D pack the central axis of the trimer on the cytosolic side.

Detailed analysis of the intermolecular interactions that stabilize this trimeric structure revealed an assembly mechanism distinct from that of canonical AQP tetramers (*5*) (fig. S5). Detailed analysis of the oligomer interface suggested that the arrangement of helices between protomers in the trimer structure of AQP11 differs significantly from that in the classical AQP tetramer structure. In canonical AQPs, the interface is formed between helices H1 and H2 of one protomer and helices H4 and H5 of an adjacent protomer (fig. S5B). In contrast, the AQP11 interface involves helices H0 and H2 of one protomer facing helices H4 and H5 of its neighbor, creating a characteristic trimer interface (Fig. 2A and fig. S5A). This difference in the helix arrangement is thought to contribute to the formation of the distinct oligomeric structures of the two AQPs. Specifically, hydrophobic interactions are observed between H0 and H4 on the ER luminal side and between H0 and H6 on the cytoplasmic side (Fig. 2B). The central portion of H0, however, does not form distinct interactions with the adjacent protomer and its surface is exposed to the lipid bilayer (Fig. 2B). The core of the AQP11 oligomeric interface is defined by extensive hydrophobic interactions between H2 and H4 (Fig. 2C). Remarkably, H1, which plays a critical role in the tetrameric assembly of canonical AQPs (fig. S5B), plays no role in forming the AQP11 trimer. The central axis of the trimer is severely constricted. On the ER luminal side, the Tyr^205^ side chain projects toward the central axis (Fig. 2D), and the cytoplasmic side is similarly occluded by hydrophobic residues on loop D (Fig. 2E). Similar to interactions observed in AQP4, interactions at the cytoplasmic oligomeric interface of the pore are formed by loop D. This suggests that loop D may be necessary for the structural integrity of the AQP11 trimer (Fig. 2E). This architecture indicates that the central oligomeric interface does not function as a permeation pathway for water or substrates.

### Detailed structural comparisons with AQP families

**Fig. 3.**
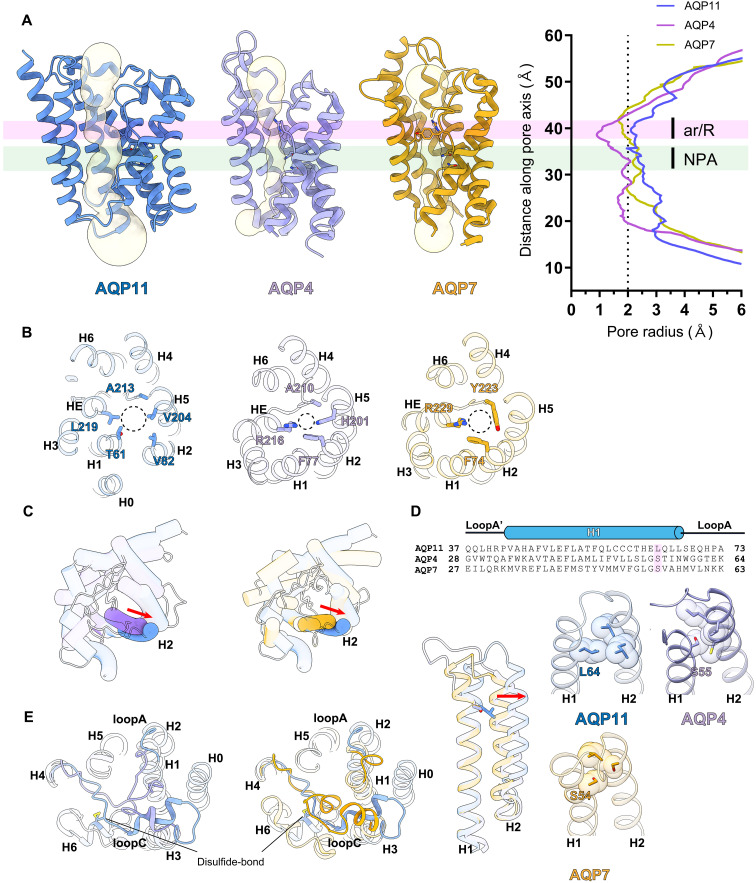
Structural comparison of other AQPs. (**A**) Pore channel profiles (left) and diameters (right) for AQP11, AQP4, and AQP7, as calculated using the HOLE2 program, are shown. AQP11, blue; AQP4, purple; and AQP7, yellow. The regions of the ar/R and NPA motifs are colored transparent pink and green, respectively. (**B**) Amino acid residues constituting the selective filter. Helical structures are shown as a ribbon model from the ER luminal or extracellular side. Residues are shown as stick models. (**C**) Distinct configuration of H2. Red arrows represent the shift of H helices. Cys^155^ and Cys^227^, forming the disulfide bond, are shown as a stick model. (**D**) Comparison of H1 among AQP11 with Leu64, AQP4 with Ser^55^, and AQP7 with Ser^54^. (**E**) Structural comparison of the loop region between AQP11 and AQP4 (left). AQP11 and AQP7 (right). Cys^155^ and Cys^227^ forming the disulfide bond are shown as a stick model.

Each AQP protomer contains a pore, approximately 30 Å in length, that spans from the extracellular to the intracellular vestibule. The substrate selectivity of AQPs is governed by a narrow constriction known as the selectivity filter located at the extracellular end of the pore. In canonical AQPs, an aromatic/arginine (ar/R) motif is the central determinant of this selectivity. For strictly water-permeable channels like hAQP4, this filter is composed of four highly conserved residues (Phe^77^, His^201^, Ala^210^, and Arg^216^). Critically, the side chain of the histidine residue extends into the pore lumen, forming a constriction smaller than 2.0 Å (Fig. 3, A and B). This constriction’s static diameter is smaller than the 2.8 Å diameter of water, which could hinder the passage of larger solutes, such as glycerol. In contrast, hAQP7, an aquaglyceroporin permeable to glycerol, possesses a selectivity filter typically formed by three bulky residues (Phe^74^, Tyr^223^, and Arg^229^). In this case, the side chain of the tyrosine residue adopts an outward-facing orientation away from the pore axis. This configuration widens the constriction point to approximately 3.5 to 4.0 Å, allowing for the permeation of glycerol (Fig. 3, A and B). hAQP11 deviates from both types. It lacks both the canonical ar/R motif of water-selective channels and the characteristic set of bulky residues found in aquaglyceroporins. Instead, AQP11 has a unique amino acid motif (Thr^61^, Val^82^, Val^204^, Ala^213^, and Leu^219^) at its selectivity filter (Fig. 3, A and B). The conserved Arg residue in AQP4 and AQP7 becomes Leu^219^ in AQP11. This motif creates a wider pore lumen that is significantly more hydrophobic than other AQPs. The pore diameter of AQP11 is 4 Å even at its narrowest point, considerably larger than that of any previously characterized AQPs. Furthermore, the pores of AQP11 are wider than those of Lsi1 and TbAQP2, which have an AQP fold, and the overall electrostatic charge and lipophilicity of all channel pores are very similar (fig. S6) (*20*, *21*). The structural basis for the wide and relatively uniform radius of the AQP11 pore, which lacks a distinct constriction site, lies not only in its unique selectivity filter composition but also in the specific positioning of H2. Structural alignment with AQP4 and AQP7 reveals that while most transmembrane helices superimpose well, H2 in AQP11 is shifted outward by approximately 4.5 Å, an extremely large value (Figs. 1E and 3C). Sequence alignments suggest a reason for this displacement. A highly conserved serine or glycine residue with a small side chain is typically found on the extracellular side of H1 in most AQPs; AQP4 has Ser^55^ and AQP7 has Ser^54^ (Fig. 3D). In AQP11, this position is substituted by a bulkier leucine (Leu^64^). We found that the side chain of Leu^64^ creates steric hindrance, displacing the adjacent H2 outward and consequently expanding the overall radius of the pore (Fig. 3D).

Extracellular loops are implicated in the substrate selectivity of AQPs. Notably, water-selective AQPs and aquaglyceroporins adopt distinct loop conformations that are conserved within their respective subfamilies. Structural superposition of AQP11 and AQP4 reveals that loop A of AQP11 is significantly shorter than that of AQP4, suggesting that its primary role is to anchor the transmembrane helices to the membrane (Fig. 3E, left). In contrast, a pronounced structural divergence is observed in loop C. In AQP4, loop C is stabilized through interactions with the long loop A, adopting a conformation where a portion of the loop dips into the water permeation pore. Conversely, loop C in AQP11 forms no clear interactions with loop A; instead, it traverses the protomer, bridging distant transmembrane helices. Notably, our study reveals that AQP11 forms a previously unreported disulfide bond between Cys^155^ on loop C and Cys^227^ on loop E (Fig. 3E and fig. S1H). This covalent linkage in the extracellular domain may contribute to the proper folding and structural stability of AQP11. AQP11 protomers forming trimers have fewer stabilizing interactions, whereas the canonical AQP protomers are all stabilized by forming a rigid tetramer. Therefore, AQP11 may need to have a stabilizer, such as a disulfide bond. Comparing AQP11 with AQP7, a representative aquaglyceroporin, further confirms the unique architectures of loop A and loop C in AQP11 (Fig. 3E, right), differing from those in both water-selective AQPs and aquaglyceroporins.

In addition to the selectivity filter, the AQP family is characterized by two highly conserved NPA motifs (fig. S7) (*13*). These motifs perform the critical dual functions of properly orienting water molecules during transit and preventing proton permeation. In AQP11, the first NPA motif is substituted with an NPC motif. A structural overlay of the NPA/NPC regions from representative AQPs, however, reveals that their three-dimensional structure is remarkably well-conserved, regardless of the substitution (fig. S8A). This structural similarity suggests that the AQP11 pore can transport substrates like water and glycerol, much like other AQPs. Nevertheless, the cysteine residue (Cys^101^) of the NPC motif appears to have a specific impact on the H6 positioning. A comparison of the AQP4 and AQP11 structures shows that H6 in AQP11 is shifted outward by approximately 6 Å relative to AQP4 (fig. S8, B and C). We postulate that this displacement arises from steric hindrance exerted by the Cys^101^ side chain, which physically pushes H6 away from the pore axis. This model is consistent with previous reports that mutations of Cys^101^ alter water permeability (*14*), providing strong support for the functional importance of this residue in AQP11.

### Functional analysis

While the physiologic function of AQP11 is not yet fully elucidated, previous studies suggested that it is water permeable, like other AQPs. To experimentally validate its function as a water channel, we purified AQP11 protein using DDM and reconstituted it into liposomes. The water transport activity of these proteoliposomes was then measured using a stopped-flow assay. The results showed that liposomes reconstituted with AQP11 exhibited a significantly higher water permeation rate in response to an osmotic gradient when compared to protein-free control liposomes (fig. S9, A to C, and table S2). In addition to measuring water permeability, our AQP11-liposome assay confirmed a significantly higher level of glycerol permeation compared to protein-free control liposomes (fig. S9, D to F, and table S2), consistent with previous reports showing that AQP11 overexpression in adipocytes significantly enhances glycerol permeability (*8*). This experimental outcome provides clear evidence that AQP11 is a functional water and glycerol permeation channel, thus corroborating the findings of previous reports (*9*, *10*). These results suggest that despite possessing an exceptionally large and hydrophobic pore, AQP11 acts as a channel for water and glycerol permeation and exhibits aquaglyceroporin function.

### Structural basis of disease-associated mutations

**Fig. 4.**
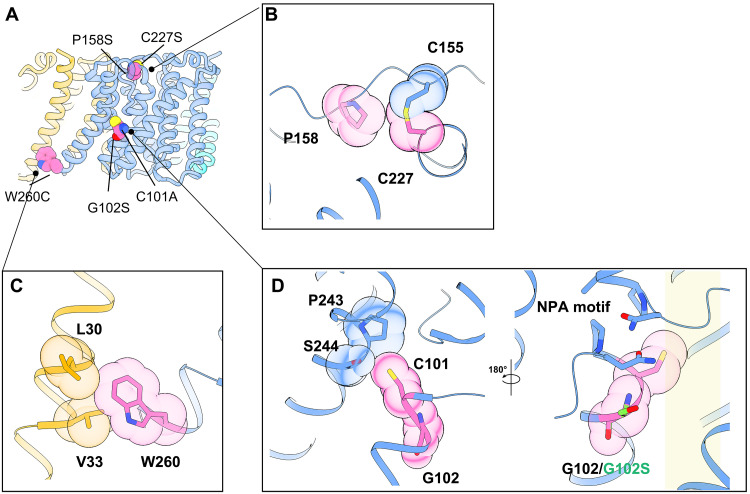
Disease-associated mutations. (**A**) Structural mapping of disease-associated mutations of hAQP11. The figure is shown as a cartoon model and colored as in Fig. 1B. Disease-related amino acid residues are shown in spherical representation. (**B**, **C**, and **D**) Side panels provide a close-up view of selected mutations. (B) Compound heterozygous mutations Pro^158^Ser is predicted to disrupt the hydrophobic interaction disulfide bond formed by Cys^155^ and Cys^277^ and destabilize loop C. Cys^227^Ser is expected to disrupt the disulfide bond and destabilize receptor folding. (C) Trp^260^Cys is predicted to disrupt hydrophobic interactions at the protomer-protomer interface, then destabilize the trimeric state. (D) Cys^101^Ala is expected to reduce interactions with H6 and affect the conformation of H6, leading to trimer instability. Gly^102^Ser is located near NPA. On the basis of the Gly^102^Ser model, the hydroxyl group of serine faces the water-permeable pore, which may affect substrate permeability. The transparent yellow squares indicate substrate-permeable pores.

Our cryo-EM structure of hAQP11 provides a direct structural framework for elucidating how pathogenic mutations lead to congenital nephrotic syndrome and polycystic kidney disease. Mapping known pathogenic variants onto our structure (Fig. 4A) revealed distinct molecular mechanisms of dysfunction.

Compound heterozygous mutations, p.Pro^158^Ser and p.Trp^260^Cys, are linked to autosomal recessive bilateral cystic renal dysplasia (*22*). Our structure shows that Pro^158^, located in ER luminal loop C, acts as a linchpin, establishing the loop’s precise topology (Fig. 4B). Notably, it is located near the disulfide bond formed by Cys^155^ and Cys^227^, as described below. Replacing this rigid proline with a flexible serine would likely destabilize this critical loop. Trp^260^, on the cytoplasmic side of H6, contributes its bulky indole side chain to extensive hydrophobic interactions at the protomer-protomer interface, with Lue^30^ and Val^33^ on H0, suggesting that it is crucial for stabilizing the oligomeric assembly (Fig. 4C).

The single-nucleotide polymorphism rs2276415 (G > A) results in a glycine-to-serine substitution at position 102 (Gly^102^Ser). Multiple studies have associated this minor allele (A allele) with an increased risk for the onset and progression of chronic kidney disease, particularly in patients with type 2 diabetes (*22*, *23*). Our structural model indicates that Gly^102^ is located near the NPA motif. This substitution would orient the hydroxyl group of the serine residue toward the substrate-permeating pore, potentially altering substrate permeability (Fig. 4D).

A naturally occurring mutation in mice, Cys^227^Ser (Aqp11*^sjds/sjds^*), induces severe proximal tubule injury, vacuolization, and lethal renal failure, with a phenotype closely resembling that of AQP11 knockout mice (*24*, *25*). Our structural analysis revealed that Cys^227^, located on the extracellular/luminal loop E, forms a covalent disulfide bond with Cys^155^ on ER-luminal loop C (Fig. 4B). This bond acts as an intramolecular tether, linking two distant regions of the protein and rigidly stabilizing the overall architecture of the extracellular/luminal domain. The Cys^227^Ser mutation would disrupt formation of this bond, impairing proper protein folding and leading to a marked decrease in expression, consistent with the induction of the unfolded protein response and apoptosis observed in the mouse model (*24*, *26*, *27*).

Last, we examined the structural effects of a previously reported experimental mutation, Cys^101^Ala, which significantly impairs oligomerization and water permeability (*14*). In our structure, the side chain of Cys^101^ forms interactions with Pro^243^ and Ser^244^ on H6 within the same protomer (Fig. 4D). In contrast, the corresponding Ala^99^ in AQP4 forms fewer interactions. A structural overlay shows that in AQP11, a steric clash with the Cys^101^ side chain causes the extracellular portion of H6 to tilt by ~30°, pivoting at Pro^243^. We propose a mechanism whereby the Cys^101^Ala mutation alleviates this steric clash, allowing H6 to adopt a more canonical orientation similar to other AQPs. This conformational shift likely disrupts the normal interface with H0, however, resulting in defective oligomerization.

Together, our structural analyses demonstrate that disease-associated AQP11 mutations disrupt function through diverse mechanisms, including protein misfolding, oligomeric destabilization, and direct obstruction of the permeation pathway.

## DISCUSSION

In this study, we determined the first high-resolution structure of AQP11 using cryo-EM. AQP11 assembles into a unique homotrimer, departing from the canonical tetrameric architecture that is a defining feature of the AQP superfamily. Furthermore, we identified a previously unknown N-terminal transmembrane helix, H0, in addition to the six established helices (H1 to H6). The presence of H0 enforces a distinct membrane topology, with the N terminus in the ER lumen and the C terminus in the cytoplasm, unlike canonical AQPs with both termini in the cytoplasm. These distinctive structural hallmarks suggest that AQP11 has followed a unique evolutionary trajectory shaped by specific functional constraints (*28*, *29*). Although our structural data indicates that AQP11 forms a stable trimer, we do not completely rule out the possibility that it may adopt different oligomeric states, such as dimers or tetramers, under different physiological conditions or through interaction with other cellular components.

Detailed analysis of the AQP11 pore architecture revealed structural features indicative of its functional specificity. First, although the pore is hydrophobic, similar to conventional water-selective channels, the pore diameter is considerably wider. Second, the selectivity filter, which is involved in substrate specificity, comprises a set of amino acid residues (Thr^61^, Val^82^, Val^204^, Ala^213^, and Leu^219^) entirely different from the classical ar/R motif. Calculating the atomic pore radius from our structural model confirms the absence of the distinct constriction site observed in other AQPs; the pore diameter is maintained at greater than 4 Å along its entire length. Coupling these structural characteristics with the established ER localization of AQP11 leads to a previously undiscovered hypothesis for its physiological function. The ER is a hub for biosynthesis and metabolism, with membrane transport extending beyond water molecules (*3*). For example, H_2_O_2_ transport is required to maintain redox balance and support signal transduction. Considering that the signaling molecule H_2_O_2_ has a molecular radius of approximately 1.5 Å (van der Waals diameter, ~3 Å), the wide and hydrophobic pore of AQP11 may be optimized not only for water permeation, but rather for the transport of larger molecules like H_2_O_2_. H_2_O_2_ permeation has been demonstrated in HeLa cells expressing the HyPer sensor (*12*). Of course, the existence of an even more optimal substrate cannot be ruled out, and further experiments are needed to address this possibility.

Last, this atomic structure of AQP11, particularly the detailed arrangement of the pore-lining residues, provides a robust foundation for future functional studies. It offers a blueprint for applying structure-guided drug design. Developing specific inhibitors or activators for AQP11 would provide chemical biology tools to control its function with temporal precision. Such compounds are expected to complement genetic approaches in elucidating the roles of AQP11 in development and under specific physiologic and pathologic conditions, holding great promise for the development of previously unknown therapeutic strategies.

## MATERIALS AND METHODS

### Protein expression and purification

The construct was expressed in Expi293F cells (Thermo Fisher Scientific) cultured in Expi293 medium (Thermo Fisher Scientific) supplied with 5% CO_2_ at 37°C. hAQP11 was expressed in Expi293F cells using the Bac-to-Bac baculovirus expression system (Thermo Fisher Scientific). Baculovirus bacmid was produced by transforming DH10Bac *Escherichia coli* cells with the pFastBacMam vector. To construct the P1 virus, the bacmid was transfected to Sf9^+^ cells (cell density, 0.8 × 10^6^ cells/ml) using FuGENE HD Transfection Reagent (Promega) in Sf900-III Serum Free Medium Complete (Gibco) with penicillin, streptomycin, and amphotericin (Fujifilm Wako) for 5 days at 27°C. To make the P2 virus, 1/100 vol of P1 virus was added to 1.2 × 10^6^ cells/ml of Sf9^+^ cells and amplified for 3 days at 27°C. hAQP11 was expressed with the P2 virus at a cell density of approximately 3 × 10^6^ cells/ml. After 24 hours, 5 mM valproic acid (Fujifilm Wako) was added to the cells, which were then transferred to 30°C and further cultured for 48 hours. The transfected cells were harvested, washed with phosphate-buffered saline, and stored at −80°C until use.

AQP11-expressing cells were suspended with lysis buffer [20 mM Hepes (7.4), 0.5 mM EDTA, 1 mM phenylmethylsulfonyl fluoride, and 20% glycerol], then homogenized using a Potter-Elvehjem homogenizer with 10 strokes. Cell debris was removed by centrifuging at 1000*g* for 5 min. The supernatant was ultracentrifuged at 30,000 rpm at 4°C for 35 min. The membrane fraction was resuspended in solubilization buffer [50 mM Hepes (7.4), 300 mM NaCl, 1% DDM, and 10% glycerol], and then incubated at 4°C for 1 hour. Insoluble materials were removed by ultracentrifugation. The supernatant was collected and subjected to affinity chromatography with TALON Co^2+^ resin (Clontech), and AQP11 was eluted using elution buffer [20 mM Hepes (7.4), 150 mM NaCl, 0.01% LMNG, 5% glycerol, and 200 mM imidazole]. Eluted protein was dialyzed to remove the imidazole and fused green fluorescent protein was cleaved by 3c protease (in-house). Last, AQP11 was further purified by SEC on a Superose 6 Increase column (Cytivia) in SEC buffer [20 mM Hepes (7.4), 150 mM NaCl, and 0.003% LMNG]. The peak fractions were collected and concentrated to 8 mg/ml for cryo-EM analysis.

### Cryo-EM sample preparation and data collection

The purified hAQP11 was loaded onto glow-discharged Quantifoil holey carbon grids (R1.2/1.3, Au, 300 mesh), which were blotted for approximately 2 s at 4°C and plunge-frozen in liquid ethane using a Vitrobo Mark IV (Thermo Fisher Scientific). Data collection was performed on a JEM-Z320FHC electron microscope (JEOL, Japan) cooled by liquid nitrogen at 300 kV, equipped with a cold field-emission gun and an in-column energy filter using a zero-loss slit width of 20 eV. All images were recorded on a K3 Summit direct electron detector camera (Gatan) operated in electron counting mode. SerialEM 4.2 (*30*) was used for automatic data acquisition. The dose rate was limited to 12.0 e−/pixel/s, and the exposure time was 2.6 s, fractionated into 50 frames without a pre-dose delay. The refined pixel size and the accumulated dose for each sample of AQPs are summarized in table S1.

### Data processing

All image processing was performed using RELION 4.0.1 (*31*) and cryoSPARC version 4.7.1 (*32*). The stacked frame movies were subjected to motion correction using MotionCor2 in RELION 4.0.1 (*33*). Contrast transfer function (CTF) estimation was performed with the cryoSPARC patch CTF algorithm. Micrographs with an estimated resolution worse than 4 Å were removed. Initial particles were picked from the yielded micrographs by the blob picker using fine 2D class averages in cryoSPARC. The initial particles were subjected to multiple rounds of 2D classification and ab initio reconstruction. To increase the number of side views, we performed Topaz picking and added them to the particle set before conducting ab initio calculations. After performing heterogeneous refinement using multiple reference maps generated from ab initio reconstruction, the classified particles were subjected to nonuniform refinement in cryoSPARC (*34*). These refined particles were then imported to RELION using the PyEM algorithm (https://doi.org/10.5281/zenodo.3576630), followed by 3D refinement, Bayesian polishing (*35*), and 3D classification without alignment in RELION. The classified particles were imported back to cryoSPARC and subjected to nonuniform refinement and global CTF refinement. Map resolutions under the refined pixel sizes were estimated using the gold-standard Fourier shell correlation with the 0.143 criterion (*36*) in cryoSPARC (fig. S1). Local resolution was estimated in cryoSPARC.

### Model building and refinement

Model building and refinement were carried out using a predicted structure from the AlphaFold Protein Structure Database (AF-Q8NBQ7-F1-v4) as the starting model (*37*), which was fitted into the obtained map using UCSF ChimeraX (*38*). The draft model was refined through iterative real-space refinement in Phenix (*39*) and manual refinement in Coot (*40*). Final map model validations were carried out using Molprobity in Phenix.

### FSEC analysis

AQP11 wild type and AQP4-GFP or mutants were expressed in Expi293F cells using a PEI transient expression system. The cells were solubilized in solubilization buffer [20 mM Hepes-Na (7.4), 300 mM NaCl, 5% glycerol, 1% DDM, 1% LMNG, 1% GDN, 1% DM, or 1% OG] for 30 min. The debris was removed by ultracentrifugation at 200,000*g* for 10 min. The supernatant was loaded onto an equilibrated Superose6 increase 10/150 GL column with running buffer [20 mM Hepes-Na (7.4), 150 mM NaCl, and 0.01% LMNG].

### Reconstitution of AQP11 into proteoliposomes

POPC lipids in chloroform were dried using nitrogen gas, freeze-dried under vacuum, and rehydrated with buffer containing 20 mM tris (pH 7.5) and 150 mM NaCl, followed by sonication. The POPC solution was diluted to 1 mg/ml with 0.1% DDM. The pooled DDM-solubilized AQPs were diluted to 0.1 mg/ml. The AQP11-monomer: POPC weight ratios were ~1:500 for mixing. Bio-Beads SM-2 resin was added, and the mixture was incubated overnight at 4°C with constant rotation. The mixture, except for the Bio-Beads, was then ultracentrifuged at 100,000*g* for 20 min. The collected pellet was suspended and sonicated in buffer containing 20 mM tris (pH 7.5) and 150 mM NaCl. Empty liposomes were made without AQPs. To measure the concentrations of proteins and phospholipids in the proteoliposomes and empty liposomes, SYPRO Ruby stain (Thermo Fisher Scientific) was applied to sodium dodecyl sulfate-polyacrylamide gel electrophoresis gels.

### Stopped-flow analysis

The 20-μl reconstituted samples diluted up to 1750 μl with buffer containing 20 mM tris (pH 7.5) and 150 mM NaCl were used for measurements carried out on a SX20 stopped-flow spectrometer (Applied Photophysics). Measurements under hyperosmotic conditions were obtained by mixing diluted samples and the same volume of buffer containing 20 mM tris (pH 7.5), 150 mM NaCl, and 200 mM sucrose, resulting in an outer buffer containing 20 mM tris (pH 7.5) and 150 mM NaCl and 100 mM sucrose, leading to an inwardly directed sucrose gradient. Another measurement was obtained by mixing diluted samples with an equal volume of buffer containing 20 mM tris (pH 7.5), 150 mM NaCl, and 200 mM glycerol, resulting in an outer buffer containing 20 mM tris (pH 7.5), 150 mM NaCl, and 100 mM glycerol, thereby creating a 100 mM inwardly directed glycerol gradient. The kinetics of vesicle shrinkage or swelling were measured by recording the change in the intensity of 90° scattered light at a wavelength of 450 nm at 20°C over time.

The change in signal intensity under sucrose hyperosmotic conditions was calculated by subtracting the averaged intensity from 0.10 to 0.14 s and normalizing it to the maximum intensity from 0.10 to 1.5 s. The time course of the intensity change under sucrose hyperosmotic conditions was plotted from 0.10 to 1.5 s and fitted to a single-exponential curve to determine the rate constant *k* using GraphPad Prism 10.4.1 (GraphPad Software). The sizes of proteoliposomes and empty liposomes were verified by a particle analyzer (ELSZ, Otsuka Electronics) at 20°C. The water permeability coefficient (*P*_f_) under hyperosmotic conditions was calculated according to the following equation as previously described (*41*): *P*_f_ = (*k* × *V*_0_)/(Δ*C* × *V*_w_ × *A*), where *V*_0_ is the volume of the liposome sample, *A* is the surface area of the liposome sample, Δ*C* is the osmotic gradient, and *V*_w_ is the molar volume of water.

The change in signal intensity under glycerol gradient conditions was calculated as the difference between the minimum (0.10 to 10 s) and maximum (0.10 to 10 s) values, normalized to the maximum. The time course of the intensity change under glycerol gradient conditions was plotted from 0.10 to 10 s. The glycerol permeability (*P*_gly_) was calculated from the following equation as previously described (*41*): *P*_gly_ = 1/[(*S*/*V*) × τ], where *S*/*V* is the surface-to-volume ratio and τ is the exponential time constant fitted to the swelling phase of the light-scattering time course.
