# Supplementary material for: Cryo-EM structure of human AQP11 reveals a trimeric architecture with a large pore
Source: Sci Adv. 2026 Jan 30;12(5):eaeb5769. doi: 10.1126/sciadv.aeb5769 (PMC12857728; doi:10.1126/sciadv.aeb5769)
Supplement: Supplementary file 1 — Figs. S1 to S9 Tables S1 and S2 [file sciadv.aeb5769_sm.pdf]

Science Advances
AAAS

Supplementary Materials for

Cryo-EM structure of human AQP11 reveals a trimeric architecture with a large pore

Shota Suzuki *et al.*

Corresponding author: Yoshinori Fujiyoshi, yoshi.cesp@tmd.ac.jp

Sci. Adv. **12**, eaeb5769 (2026)
DOI: 10.1126/sciadv.aeb5769

This PDF file includes:

Figs. S1 to S9
Tables S1 and S2

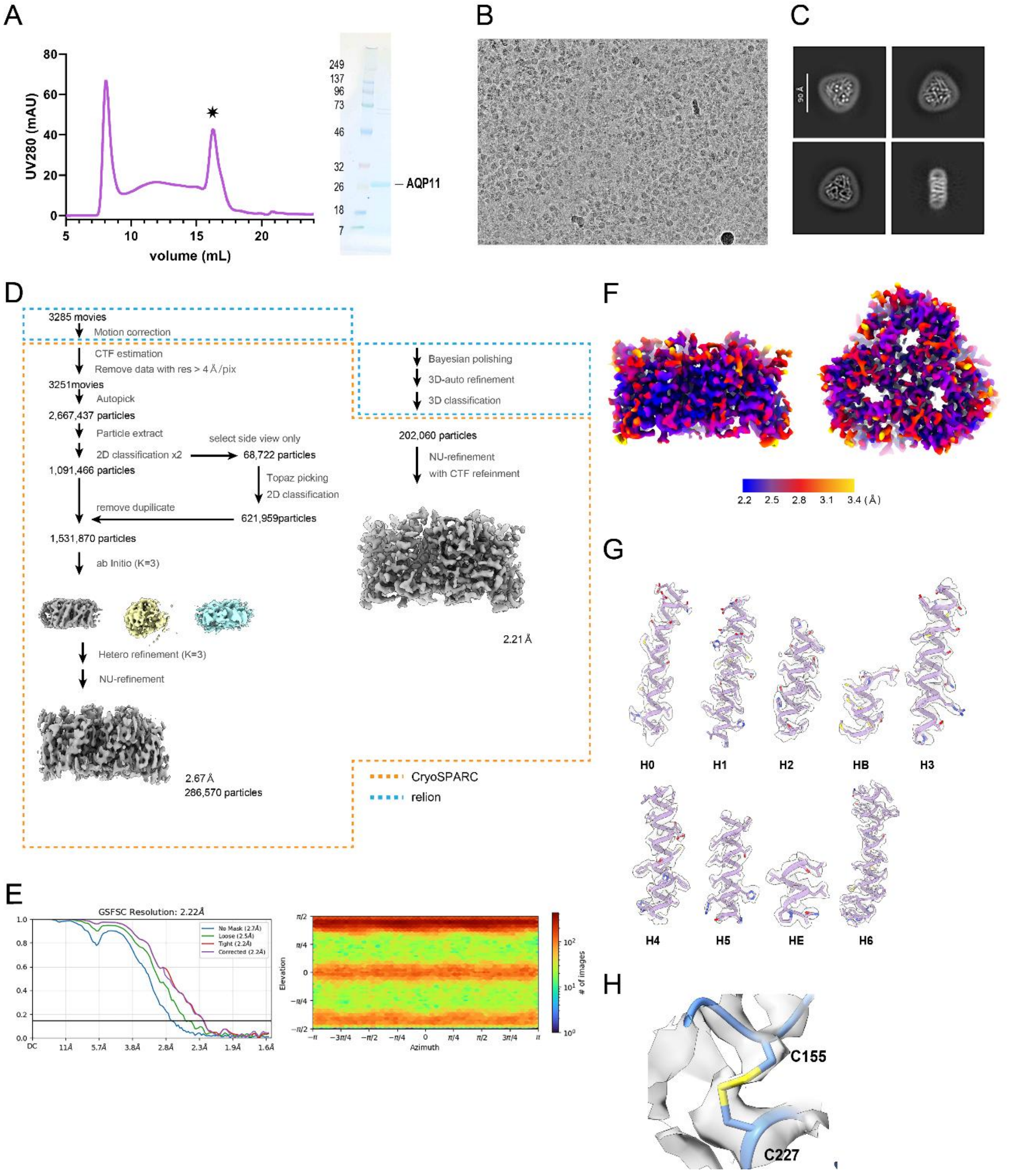

Fig. S1. Sample preparation and structural analysis of human AQP11

(A) Representative elution profile of AQP11 on a Superose 6 Increase 10/300 column and SDS-PAGE of the peak fraction (symbol). **(B, C)** Representative cryo-EM micrograph and 2D class averages. **(D)** Workflow for cryo-EM data processing of AQP11. **(E)** Gold-standard Fourier shell correlation (GSFSC) curves and angular distribution plots for AQP11. **(F)** Local resolution analyses of AQP11. **(G)** Cryo-EM density maps and models are also shown for H0-H6, HB, and HE. **(H)** Cryo-EM density of the disulfide bond between Cys155 and Cys227.

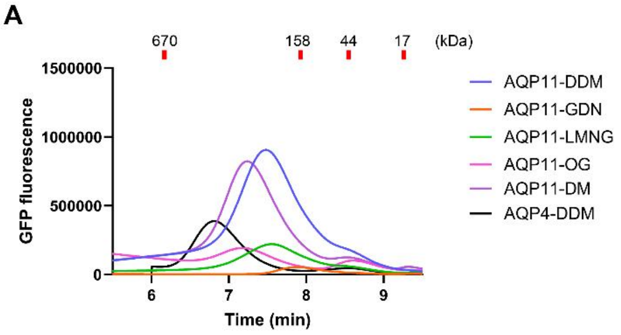

B

Sample	Elution Time (min)
AQP11-DDM	7.5
AQP11-LMNG	7.6
AQP11-GDN	7.8
AQP11-DM	7.2
AQP11-OG	7.2
AQP4-DDM	6.8

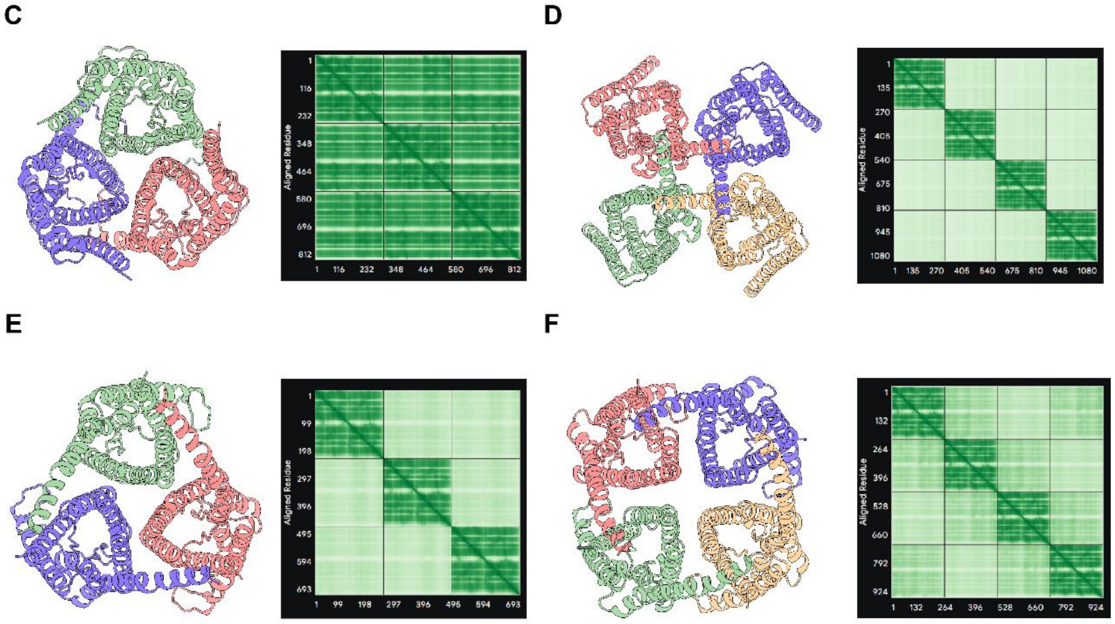

Fig. S2. Oligomeric state of AQP11

(A) Fluorescence-detection size-exclusion chromatography (FSEC) profiles of several detergents solubilized AQP11-GFP and the tetrameric control DDM solubilized GFP-AQP4. The red mark at the top indicates the position of the Gel Filtration Standard (Bio-Rad). **(B)** Elution peak time of AQP11 solubilized with different detergents. (C-F) The oligomeric state of AQP11 was predicted using the AF3 server by submitting the amino acid sequence with a specified copy number. **(C)** Full-length AQP11 modeled as a trimer (3 copies). **(D)** Full-length AQP11 modeled as a tetramer (4 copies). **(E)** An AQP11 construct lacking the N-terminal helix H0 (AQP11-ΔH0), modeled as a trimer (3 copies). **(F)** The AQP11-ΔH0 construct is modeled as a tetramer (4 copies). For each panel, the predicted structure is shown as a ribbon model on the left, with individual protomers colored differently. The corresponding Predicted Aligned Error (PAE) plot is shown on the right.

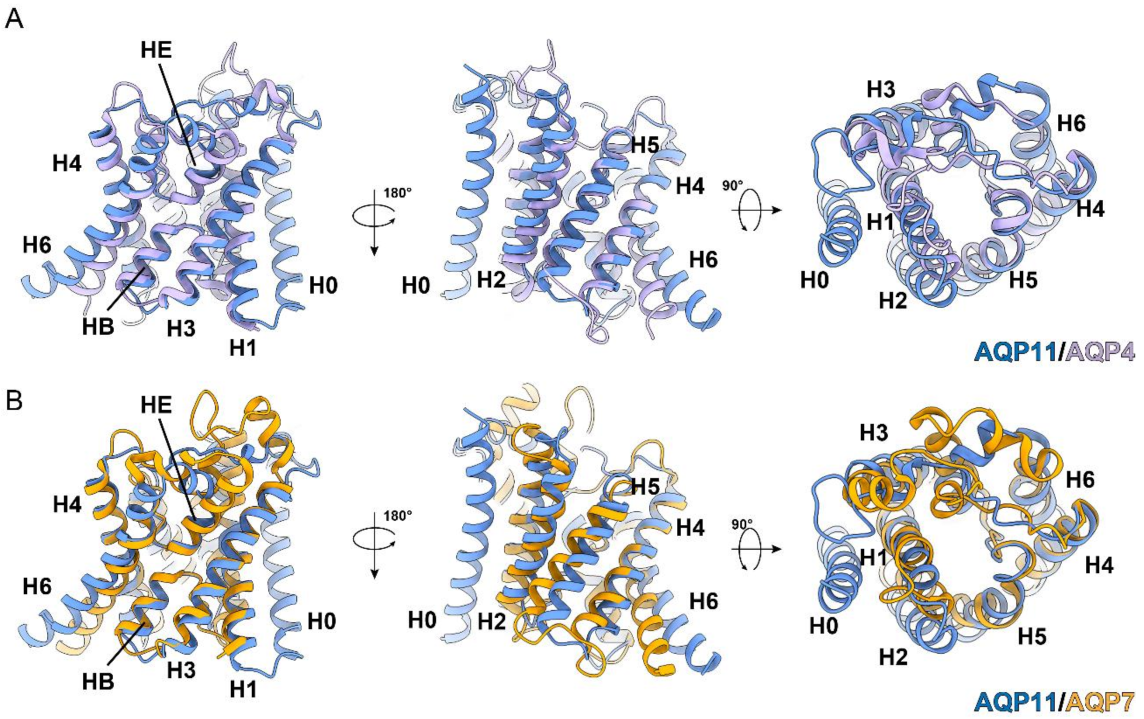

Fig. S3. Structural comparison of canonical AQPs

Structural overlays illustrate the high degree of fold conservation between the AQP11 protomer and representative canonical aquaporins. In all panels, AQP11 is colored light blue. **(A)** Overlay of AQP11 with the human water-specific channel AQP4 (shown in light purple, PDB ID: 3GD8). **(B)** Overlay of AQP11 with the human aquaglyceroporin AQP7 (shown in orange, PDB ID: 6QZI). The structures are shown in side view (left), rotated by 180° (middle), and in an extracellular top view (right).

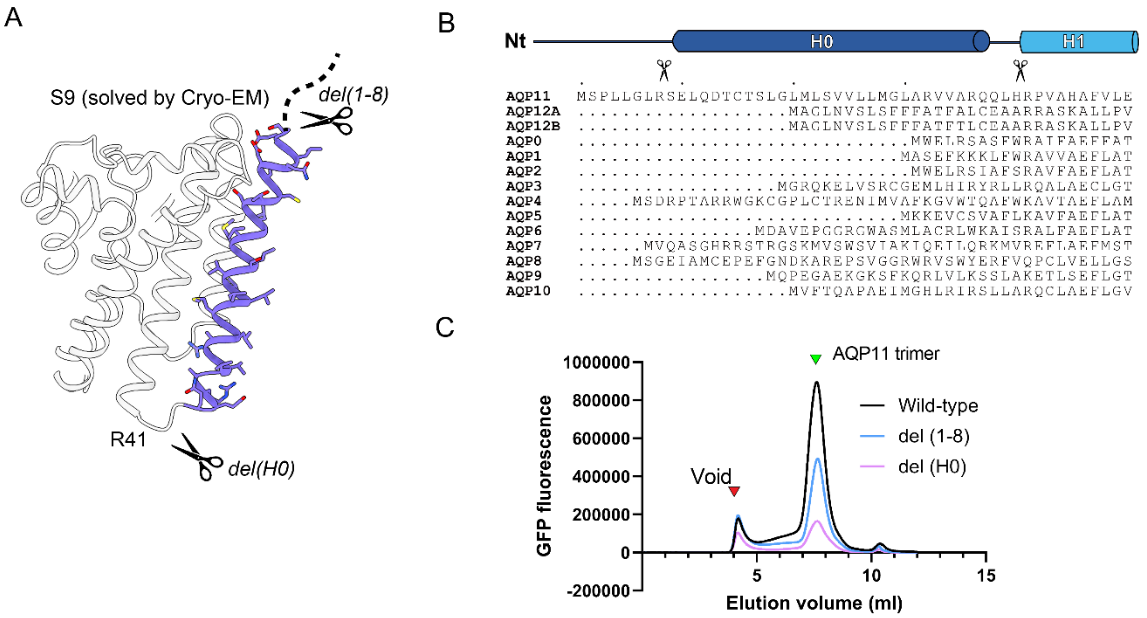

Fig. S4. The importance of H0

(A) Structure of the AQP11 protomer. H0 is highlighted in purple. **(B)** Sequence alignment of the N-terminus of AQPs. The cartoon shows the location of the helices in AQP11. Use scissors to indicate deletion points. **(C)** Analytical GFP fluorescence chromatography profile of AQP11 wild type- or its mutants-expressing cells solubilized by DDM.

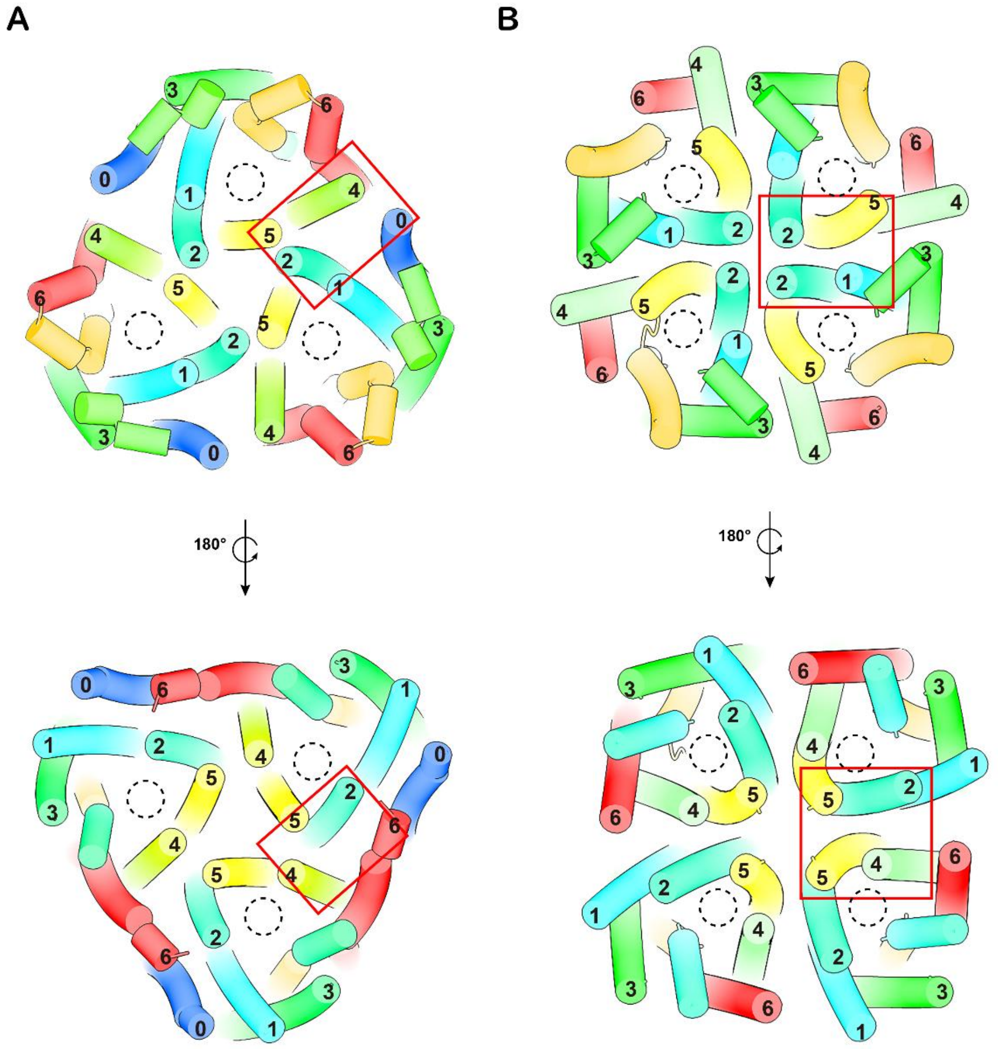

Fig. S5. Different oligomeric states of AQPs

The trimeric and tetrameric modeled structures of hAQP11 **(A)** and hAQP4 **(B)** are shown as a ribbon representation from the ER lumen or extracellular view (upper) and cytosolic view (lower). Each model is highlighted in rainbow colors. The red squares indicate the transmembrane region involved in trimer and tetramer formations. The numbers indicate helix numbers. Black dot circles indicate the water channel pore of each protomer.

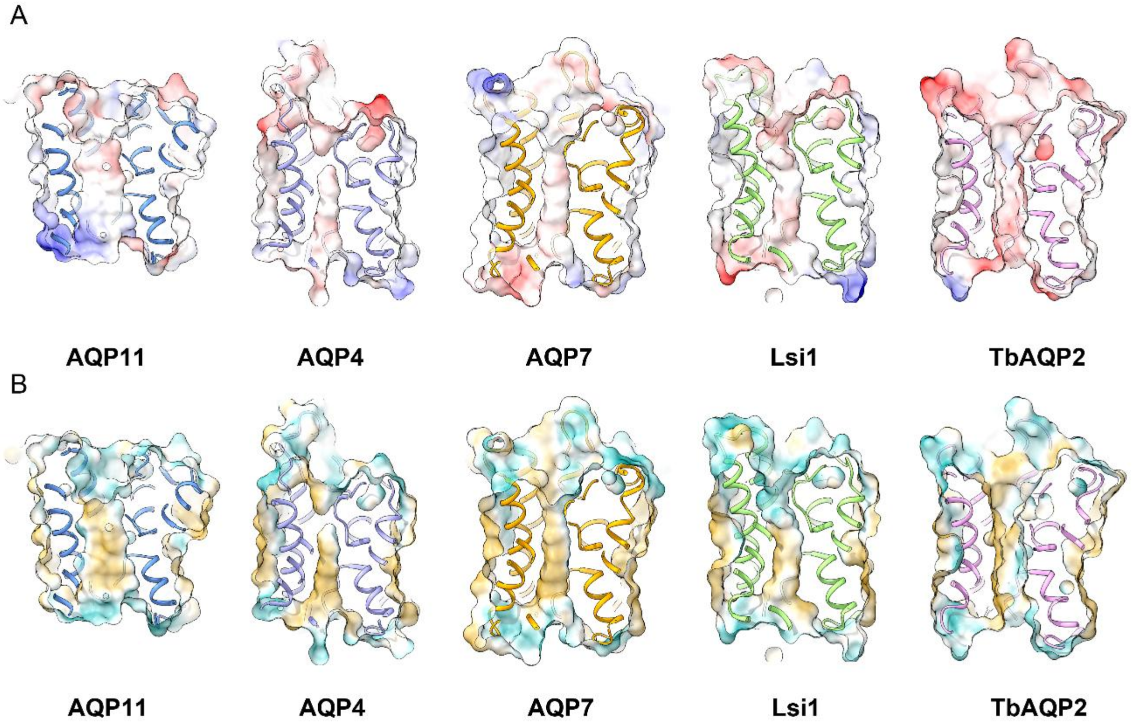

Fig. S6. Structural comparison of several AQP fold channels

Structural comparison and cross sections of different AQP fold channels of AQP11, AQP4 (PDB 3GD8), AQP7 (PDB 6QZI), Lsi1 (PDB 7CJS), and TbAQP2 (PDB 8JY7). Electro surface potential **(A)** and hydrophobicity **(B)** are shown. Cartoon models are superimposed on surface models. Displays are shown with the front section of the channels omitted for clarity.

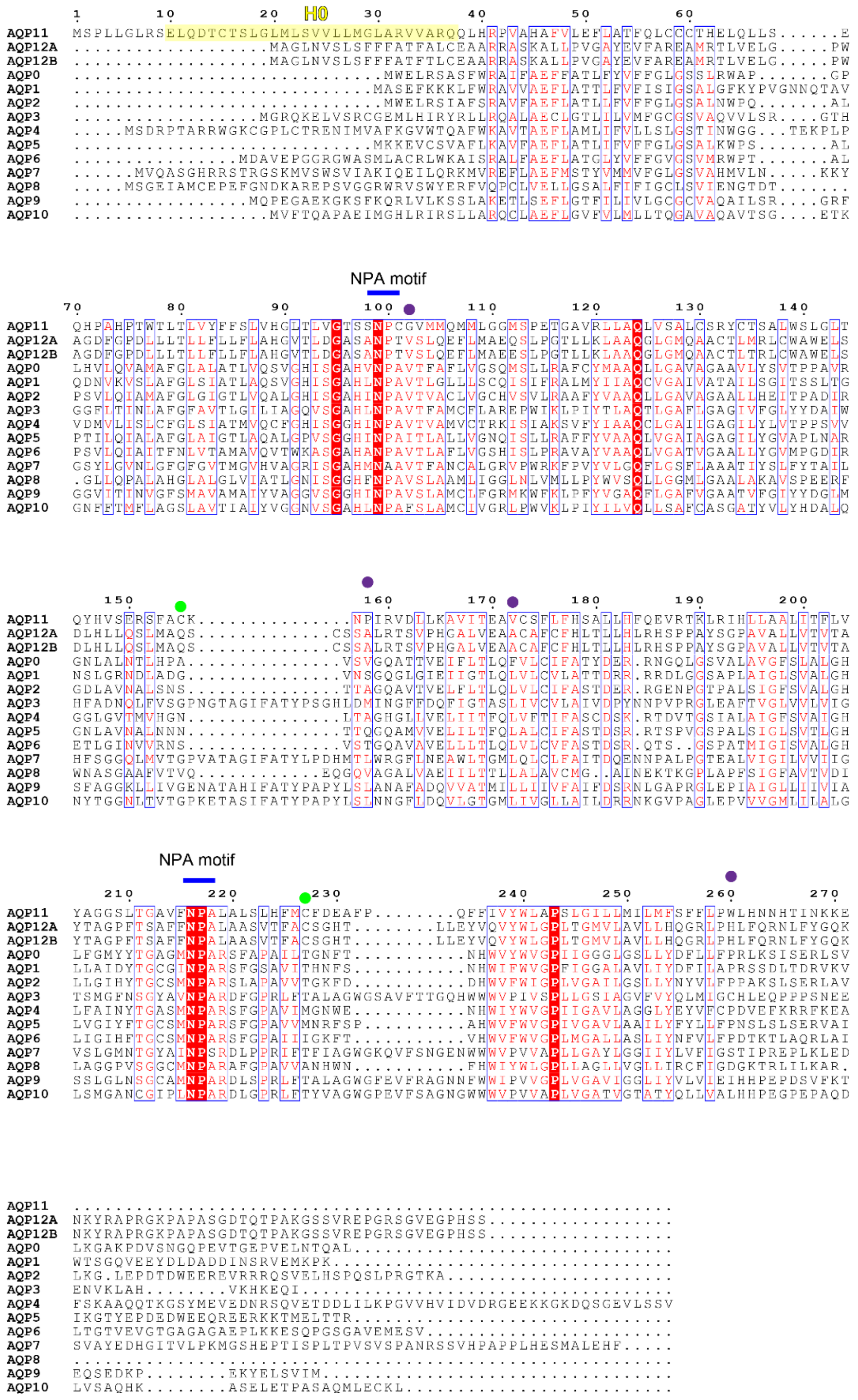

Fig. S7. Sequence comparison of aquaporin proteins.

Comparison of amino acid sequences in the AQP family. Alignment was rendered using ESPript 3.0 with default settings for similarity calculations. Identical (white letters filled with red color) and similar (red letters with blue box) amino acids are denoted. Green circles indicate cysteine residues forming disulfide bonds, and purple circles indicate disease-related mutation sites.

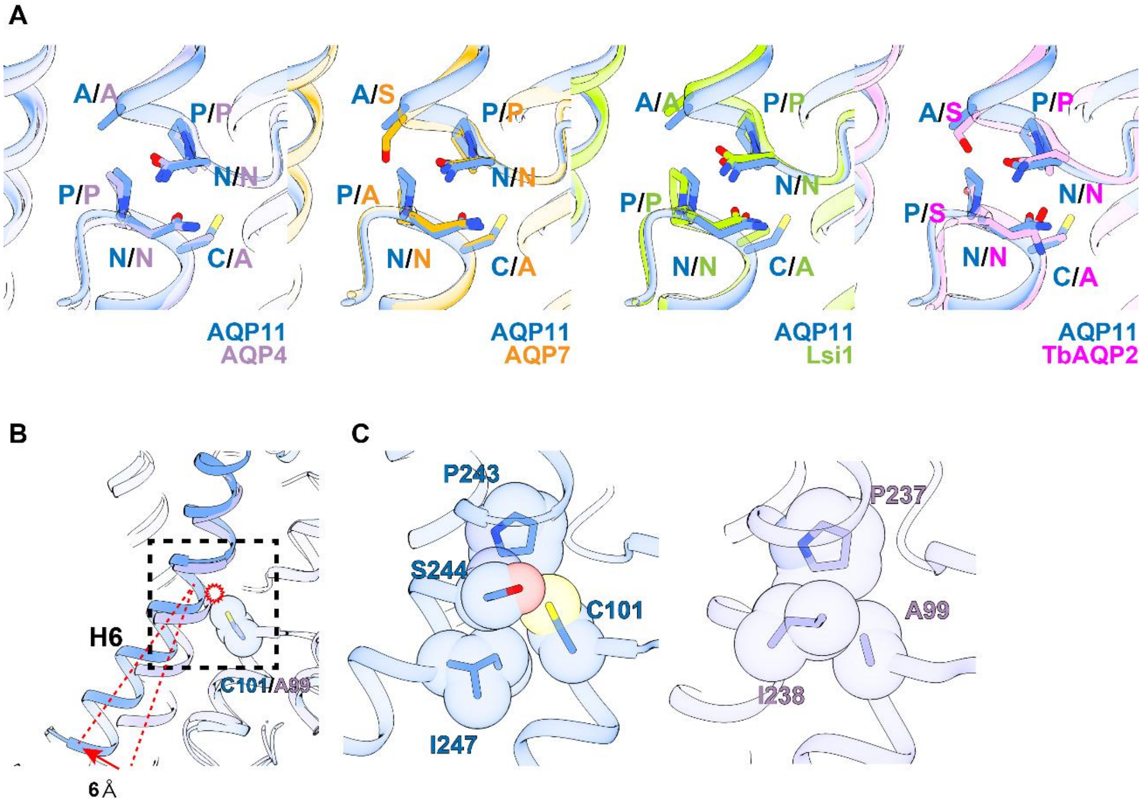

Fig. S8. Structures of the NPA(C)/NPA motifs.

(A) Cartoon representations of the HB and HE of hAQP11, hAQP4 (purple, PDB: 3GD8), AQP7 (orange, PDB: 6QZI), Lsi1 (green, PDB: 7CJS), and TbAQP2 (pink, PDB: 8JY7). NPA/NPA motifs and residues are drawn as stick models. **(B)** Superposition of H6 on AQP11 and AQP4, with angular differences indicated by red arrows. The side chain of Cys101 in AQP11 is larger than the corresponding alanine in AQP4, physically pushing H6 outward. **(C)** Detailed intramolecular interactions between the NPA motif and H6 residues.

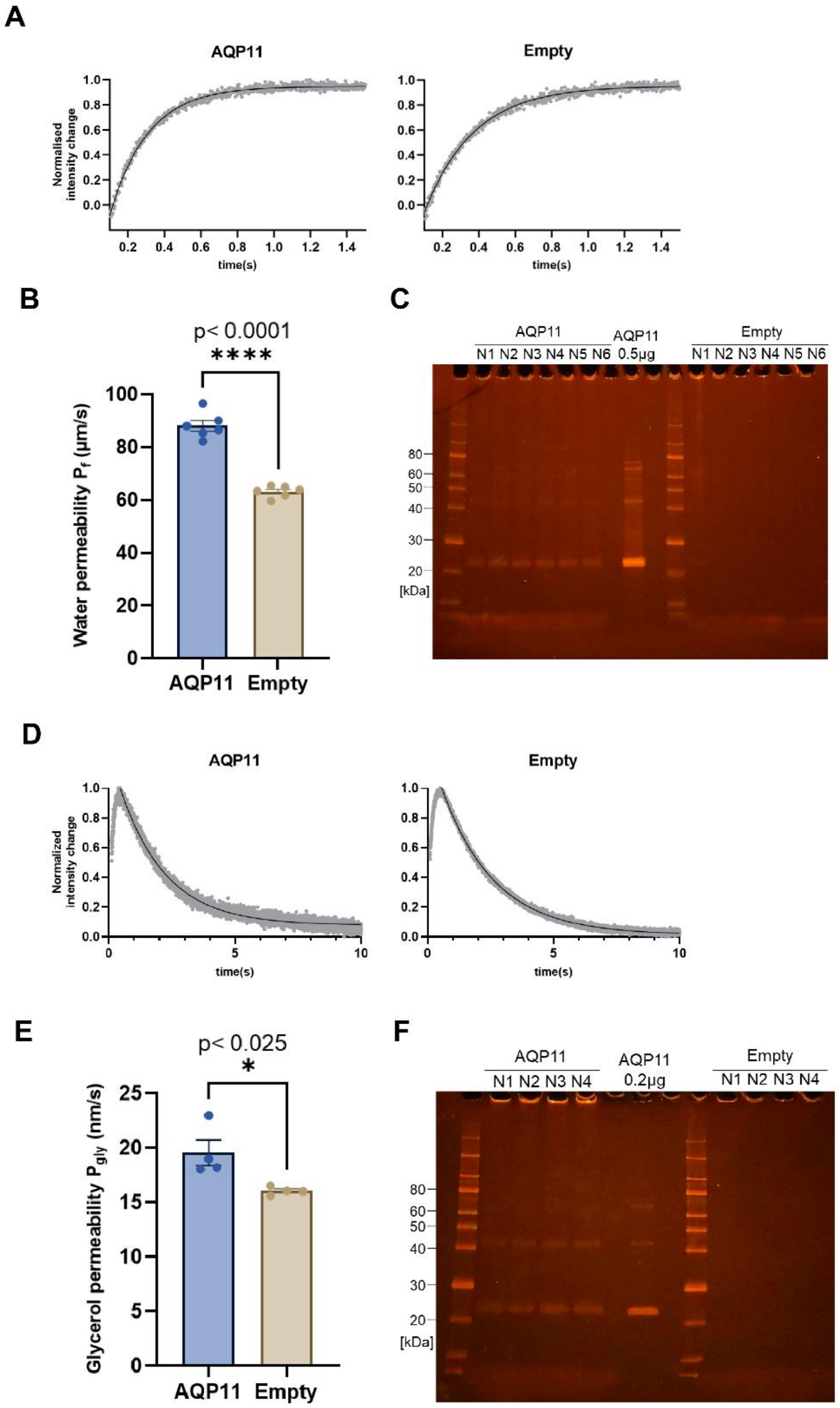

Fig. S9. **Functional analysis of human AQP11 proteoliposomes**

(A, B) Stopped-flow measurements under sucrose hyperosmotic conditions for hAQP11 proteoliposomes and empty liposomes. Average water permeability (P_f) values ± SEM and individual data points are plotted (n = 6 independently reconstituted liposome samples from the same purified proteins). Data were statistically analyzed using Student's two-tailed unpaired *t*-test. P-value is

indicated. ****P < 0.0001. Representative time courses of normalized intensity change and fitted curves are shown (A). **(C)** SYPRO Ruby staining of hAQP11 proteoliposomes and empty liposomes. Independently reconstituted liposome samples (n = 6) from the same purified proteins were stained once. **(D, E)** Stopped-flow measurements under glycerol gradient conditions for hAQP11proteoliposomes and empty liposomes. Averaged glycerol permeability P_{gly} values ± SEM and individual data points are plotted (n = 4 independently reconstituted liposome samples from the same purified proteins). Data were statistically analyzed using Student's two-tailed unpaired t-test. *P* values are indicated. **P* < 0.05. Representative time courses of normalized intensity change and fitted curves are shown (D). **(F)** SYPRO Ruby staining of hAQP11 proteoliposomes and empty liposomes. Independently reconstituted liposome samples (n = 4) from the same purified proteins were stained once.

Table S1 | Cryo-EM data collection, refinement, and validation statistics

	hAQP11 (EMDB: EMD-65443) (PDB 9VXW)
**Data collection and processing**	
Magnification	60K
Voltage (kV)	300
Electron exposure (e–/Å^2)	50
Defocus range (μm)	-0.7 to -1.5
Pixel size (Å)	0.79
Final particle images (no.)	202,060
Map resolution (Å)	2.22
FSC threshold	0.143
Map resolution range (Å)	2.2-3.4
**Refinement**	
Model resolution (Å)	2.4
FSC threshold	0.5
Model resolution range (Å)	n/a
Map sharpening *B* factor (Å^2)	-61.6
Model composition	
Non-hydrogen atoms	1,979
Protein residues	254
Ligands	1
RMS deviations	
Bond lengths (Å)	0.003
Bond angles (°)	0.490
Validation	
MolProbity score	2.09
Clashscore	6.25
Poor rotamers (%)	4.17
Ramachandran plot	
Favored (%)	0
Allowed (%)	3.97
Disallowed (%)	96.03

Table S2 | Analyses of POPC liposomes.

Water permeability	AQP11 LPR 500 (n=6)	Empty (n=6)
Diameter of liposomes (nm)	193.3 ± 2.128	185.1 ± 2.868
k (1/s)	4.919 ± 0.0647	3.686 ± 0.0387
P_f (µm/s)	88.07 ± 1.9772	63.16 ± 0.8776

Glycerol permeability	AQP11 LPR 500 (n=4)	Empty (n=4)
Diameter of liposomes (nm)	212.4 ± 2.410	203.0 ± 0.966
τ (s)	1.825 ± 0.0851	2.108 ± 0.0197
P_{gly} (nm/s)	19.55 ± 1.1601	16.05 ± 0.1934
